# Snapshot of the Use of Urban Green Spaces in Mexico City during the COVID-19 Pandemic: A Qualitative Study

**DOI:** 10.3390/ijerph18084304

**Published:** 2021-04-18

**Authors:** Carolina Mayen Huerta, Gianluca Cafagna

**Affiliations:** 1 School of Geography, Earth and Atmospheric Sciences, University of Melbourne, Level 1, Bouverie Street, Melbourne, VIC 3010, Australia; 2 World Bank Group, Washington, DC 20433, USA; gcafagna@worldbank.org

**Keywords:** urban green spaces, well-being, photography, UGS, factors of use

## Abstract

The present qualitative research explores the factors that have influenced the use of urban green spaces (UGS) in Mexico City during the COVID-19 pandemic and the implications of their usage on residents’ well-being. This study was conducted using a combination of solicited audio and written diaries, photography, and in-depth interviews with 16 participants, aged 22 to 58. The article provides a critical reflection on the incentives and deterrents to the UGS use of participants while social distancing measures were in place. The results show that in Mexico City: (1) participants’ lack of access to UGS has hampered their use, mainly among those of low-income neighborhoods; (2) UGS size did not directly impact participants’ UGS use during the pandemic; and (3) women were deterred from accessing UGS due to safety concerns related to the fear of violence. Overall, the results suggest that UGS use has served as a coping mechanism to decrease the effects of stress and isolation caused by the pandemic, increasing users’ physical and mental well-being. This study’s conclusions can help develop future citizen participation tools that are useful for resilience in urban design, as they provide interesting insights into the perceptions of residents, such as the most valued characteristics of UGS.

## 1. Introduction

Worldwide, a burgeoning number of studies have concluded that urban green spaces (UGS) provide essential benefits such as promoting physical activity, reducing stress and fatigue, improving air quality, and enhancing cities’ aesthetic value [1,2,3,4]. This final benefit directly elevates people’s sense of life satisfaction, which in turn contributes to an upsurge in their quality of life [5]. Furthermore, evidence suggests that increased UGS use positively correlates with both physical and mental health indicators among individuals and communities; this could prove beneficial in emergency circumstances such as the present COVID-19 pandemic [6,7,8]. Nonetheless, UGS use in Mexico City declined by 34% in 2020 compared to 2019 [9]. This outcome is particularly concerning because of the public health benefits associated with using UGS [10]. Moreover, it contrasts with outcomes in countries like Germany, Norway, and the United States, where UGS use has increased due to the prioritization of accessible, high-quality green spaces [11,12].

To the best of the authors’ knowledge, the limitations and incentives that may have affected UGS use in Mexico City during the COVID-19 pandemic have not yet been reported in the literature. Moreover, it remains unclear whether the association between UGS use and well-being has been altered by the social distancing regulations and risks associated with spending time outside the home. Consequently, this study has two objectives: (1) exploring the factors affecting participants’ UGS use during the COVID-19 pandemic and (2) analyzing the relationship between participants’ UGS use and well-being. Although the present study results cannot be generalized, due to its qualitative approach, they can prove useful for incentivizing conversations on how to create an adequate UGS infrastructure throughout the city and build resilience during a health crisis.

## 2. Factors Affecting the Use of UGS

In order to design UGS that people will use, urban planners need to understand how green spaces’ characteristics affect residents’ perceptions of and willingness to use them, exploring their motivations through citizen participation instruments [13]. Van Herzele and Wiedemann [14] have referred to the factors that affect UGS use as the “preconditions for use” [14]. These preconditions are related to (a) access, understood as proximity; (b) size; and (c) safety [15], and frequently appear in the literature evaluating UGS use [16,17,18]. Conversely, “satisfiers” are characteristics pertaining to quality that can incentivize or discourage use [14]. While satisfiers are not considered preconditions of use, they can heavily influence behavior patterns.

### 2.1. Access Precondition

Access to UGS is widely understood as the physical proximity or relative nearness to the spaces in question [16,19,20,21]. The opportunities and enjoyment created by UGS are restricted by geography [22]. Thus, proximity is a necessary precondition for determining which UGS are geographically accessible and how often they are visited [17,23]. The literature on UGS defines adequate access as a distance of 15–20 min walking at a moderate pace, which is thought to be the maximum distance people are willing to consider walking to reach a green space [2,24,25,26,27].

From March to June 2020, the access precondition held particular importance, as the COVID-19 restrictions severely restricted individuals’ movements. Inequitable UGS distribution grew increasingly apparent in Mexico City, where most UGS are located in the northwest part of the city [28]. In contrast, the southeast region, home to the most marginalized segments of the population, has the lowest level of green area availability. Most neighborhoods in this area lack access to UGS within the recommended walking distance of 15 to 20 min [29,30]. With mobility restrictions in place, lack of access posed an additional barrier to UGS use.

The heterogeneity in UGS access in Mexico City was previously reported by the Federal District’s Environmental Land Management Agency in 2009 [31]. Additionally, in 2017, Fernández-Álvarez [32] evaluated the availability of green space in Mexico City’s various municipalities according to their marginalization classification. He used a container approach, measuring the percentage of total green areas located in each municipality [33]. The results showed that green space availability is associated with a municipality’s socioeconomic status, implying that UGS access is inadequate in the poorest municipalities of Mexico City.

### 2.2. Size Precondition

The question of whether UGS will be used must take into consideration how much green space is considered enough to benefit from them [34,35]. According to the literature, the synergies between the benefits that UGS provide increase in correlation with the size of the space [20]. In addition, size is critical because it limits or enables specific activities and perceptions [36,37]. For instance, larger UGS have been associated with higher perceptions of restorative effects, as people experience increased contact with nature and lower noise levels [38]. Therefore, size is a precondition linked to the functionality of the space that dictates how it can be used [14]. Furthermore, evidence on UGS use indicates that, as size increases, the willingness to travel to the green space also grows [14,39,40,41].

During the pandemic, the size precondition has had significant consequences in determining the UGS use. On 20 May 2020, the mayor of Mexico City presented the Gradual Plan Toward the New Normal, which defined strategies to progressively resume social, educational, cultural, and economic activities while adhering to health safety standards [42]. The plan introduced a traffic light system and a monitoring committee to evaluate the epidemiological risks of COVID-19. The traffic light system restricts the use of public spaces, including UGS, depending on their epidemiological risk. The system classifies spaces using three scenarios: (a) red/maximum health risk, which implies restricting the use of UGS to 30 percent of their capacity; (b) yellow/intermediate health risk, which implies restricting the use of UGS to 60 percent of their capacity; and (c) green/low health risk, which implies no restrictions on the use of UGS while maintaining social distance.

This particular constraint disproportionately affected densely populated areas with access only to UGS that are small in size, being that the size of the space is directly linked to the number of likely users. Some municipalities unexpectedly opted for the full closure of smaller UGS, given the difficulties of enforcing occupancy rates. Others closed UGS to visitors and then reopened them in June 2020 at only 30 percent capacity, even when the traffic light system marked the risk as yellow/intermediate. Municipal governments made decisions about each UGS based on their size and occupancy rates, the population density of the surrounding areas, and the risk of contagion. High-income municipalities, which have more resources, were less likely to close their UGS.

### 2.3. Safety Precondition

In the context of the pandemic, safety was linked to two main dimensions. The first dimension, violence, was associated with the fear of being attacked, robbed, or assaulted while using UGS [43]. This dimension is the most frequently studied in the literature and correlates with UGS maintenance, which relates to crime perceptions [44]. In Mexico, safety in public spaces—which pertains to violence—has been especially important for women because gender violence is an ongoing problem, resulting from the country’s structural inequalities [45]. As UGS occupancy rates fell during the pandemic, perceptions that UGS were unsafe began to rise, ultimately leading to a decline in use by women [46].

The second dimension, health, relates to fears of contracting or spreading the virus and is exclusive to the pandemic circumstances. The oversaturation of spaces has become a risk factor that complicates social distancing mandates [47]. Health-related fears have reduced the use of UGS in Mexico City. For instance, in a previous study conducted in the city (*n* = 1954), 71.8 percent of respondents who stopped using UGS during the pandemic cited concerns about the risks those spaces posed for their health and anxieties about contracting COVID-19 or infecting a close relative [30].

### 2.4. Quality Satisfiers

Data shows that access, size, and safety preconditions are not the only factors affecting UGS utilization—quality features can also encourage people to take advantage of public spaces [14]. In some cases, quality can prevent UGS from fulfilling their environmental, recreational, social, and psychological roles or restrict their use to certain segments of the population [48,49,50]. Therefore, it is important to understand which features promote the enjoyment of green spaces [26,51,52,53]. Some of the many quality features that can motivate use are benches, amenities, and playgrounds; planned activities; and informal sellers or markets. In sum, UGS that are perceived to be of higher quality attract more activity and, therefore, use [54].

## 3. Study Precedent

In June 2020, a survey (*n* = 1954) was developed and administered through social networks to evaluate the links between UGS use and the mental well-being of Mexico City’s population under social distancing. The survey results showed that 33.2 percent of respondents had stopped using UGS from March to June 2020. Additionally, the decline in UGS use was associated with poorer mental well-being outcomes, with women reporting worse outcomes than men when evaluated by the short version of the Warwick–Edinburgh mental well-being scale (WEMWS) [30]. The results contrasted with those in cities like Oslo and New York, where people had increased their use of UGS during the pandemic [12]. The present study further investigates the conclusions of the June 2020 survey by analyzing the experiences of a smaller number of participants. Since this work focuses on addressing the reasons behind UGS use rather than reporting associations, having a small number of participants allowed researchers for more frequent and prolonged interactions with each participant, providing an in-depth appraisal of the motivations and disincentives for UGS use and the implications of this use for well-being. The number of participants in this study is comparable to other qualitative studies focusing on analyzing individuals’ behaviors and well-being [55,56]. Moreover, as this study was conducted seven months after the social distancing measures began (September–October 2020), it provides a different perspective from its predecessor [30], which captured the residents’ attitudes three months into the social distancing period.

## 4. Methods

The present qualitative study used a combination of solicited written and audio diaries, participant-generated photographs, and semi-structured interviews to explore UGS use’s factors and their impact on Mexico City’s residents’ well-being during the COVID-19 pandemic. This combination of methods was selected to yield introspective conversations with participants about often-overlooked topics or behaviors that are nonetheless significant to their everyday lives. Specifically, this research delves into the participants’ thoughts, feelings, and mood when using UGS during the restrictions of the pandemic, all of which are associated with their well-being

Latham [57] supports the methodological use of the “diary photograph” because it facilitates engagement with a study’s participants and helps visualize their circumstances, revealing their identities and belief systems through their practical actions. This visual method also reduces power asymmetries between researchers and participants [58], as introducing an ex-situ component minimizes researchers’ influence on participants’ behavior [59].

The combination of photography, diaries, and interviews has been used in various qualitative studies for exploring attitudes, behaviors, and self-evaluations, which are all associated with well-being [60,61]. For instance, Gibson et al. [62] applied this approach to study the experiences of 11 disabled young men transitioning into adulthood in Ontario, Canada. Similarly, Bijoux and Myers [55] combined solicited diaries, photography, semi-structured interviews, and mapping to document differences in women’s behavior, sense of attachment, connectedness, and well-being in relation to their place of residence in Auckland, New Zealand. Giritli-Nygren and Schmauch [63] used photographs as a mechanism to explore the experiences of 14 immigrant women in Sweden, highlighting their social relationships and feelings of inclusion or exclusion. Finally, in the Caprivi region of Namibia, Thomas [5] reviewed solicited diaries and photography to explore the emotional well-being of people living with HIV/AIDS.

Given the social distancing regulations in Mexico City and the effectiveness of photography in capturing obscure elements of everyday life, this combination of methods was considered the safest and most effective for the present study. It is also important to highlight that self-directed photography promotes engagement and empowers participants. This allows researchers to understand participants’ context in the face of difficulties in communication or access to the participants’ environment [55]. The latter was the case in the present study.

### 4.1. Study Participants

An invitation to the study was sent through social networks (Facebook and Twitter) to people who met a certain demographic profile in terms of place of residence, gender, income, age, and living situation. The invitation described a two-week study using photographs to explore the well-being of Mexico City inhabitants during the pandemic. Interested parties were asked to send an email describing their socioeconomic profile and characterizing their overall perception of their neighborhood’s quality. The latter was used as a proxy for establishing UGS quality in the participants’ neighborhoods and was later corroborated with simple quality-related questions during the final interview. These questions included whether the UGS nearest the participant’s home was large enough to meet the neighborhood’s basic needs and whether amenities such as adequate lighting, benches, bathrooms, running tracks, and playgrounds were present. Communication with the participants was established via email and WhatsApp. WhatsApp was chosen because it is a common means of communication in Mexico, with 77 million users [64]. All communication was conducted in Spanish.

Twenty-eight people offered to participate in the study. After reviewing their profiles, 16 participants between 22 and 58 years old were invited to contribute. The female-to-male ratio, 11 to 5, was established based on the previous survey results, which showed a more pronounced decline in mental well-being outcomes and UGS use among women in the WEMWS evaluation. Furthermore, younger age groups were more likely to be included in the study, as younger people also reported worse mental well-being outcomes, measured through the same scale [30].

It is important to note that adults above the age of 58 were not included in the study. This limiting selection bias can be explained in part because participants were selected through social media. Older adults tend to be less tech-savvy and thus less inclined to use Facebook or Twitter [65]. More studies of older adults are needed to examine why they use UGS and how UGS use affects their well-being.

The research included people living in the most marginalized neighborhoods of the city and those in neighborhoods with high UGS access levels. Lastly, this study compares the perspectives of people living alone versus people living with others. Both profiles were incorporated in the study to address the documented impact of isolation on behavioral patterns and well-being [66]. Because place, cultural background, differences in the built environment, and social behaviors can all influence individuals’ actions [67], it was crucial to explore how people with contrasting circumstances and demands perceive UGS. The diversity of the study sample enabled greater insights into the physical and physiological effects of the pandemic. The general profiles of the participants are listed in Table 1.

During the selection and consent processes, participants were given the opportunity to pose questions at any time. To adequately explain the study’s objective and instructions, an initial 20-min WhatsApp call with each participant was conducted during the final week of September 2020. During the call, the participants were given the express assurance that: (1) no identifiable information would be made public, (2) involvement in the study was completely voluntary, and (3) participants had the right to leave the study at any point. Furthermore, the contributors were asked for verbal consent to publish their photographs and excerpts from their interviews. After the study finished, all participants were informed which quotes, excerpts, and pictures were included, and signed written consent was requested.

### 4.2. Photo Journal

The photo-journaling occurred between 29 September and 22 October 2020. The beginning and end dates of each participant’s photo journal were subject to participants’ schedules. The participants were asked to share a minimum of 14 pictures (one per day), showcasing what they considered to be the most relevant experiences of their day, both positive and negative. With each photograph submission, the participants were required to include a written description or voice note about the feelings that accompanied the documented experiences as well as what was happening, where they were, and whom they were with. Since offering various means of communication has been shown to yield greater comfort and broader self-expression among participants [62], both written and voice-recorded options were accepted.

A key goal was assessing what the participants perceived as important or influential for their well-being. No specific instructions were given on what type of photographs to take, and no mention was made of ‘urban green spaces’ in the instructions. It was critical to observe whether participants included UGS or natural environments among the photographs of places they associated with positive feelings. At the end of the 14-day photo-journaling period, an in-depth interview ranging from 40 to 60 min in length was conducted with each participant. Each participant was asked about their behavioral habits during the pandemic—How often they go out? What places do they frequent the most? What type of activities do they do? Also, participants were asked for potential anxieties of going out and changes in their interactions with others. Interviews included questions about specific photographs and thoughts contained in the diaries of each participant. Furthermore, questions about the participants’ mental well-being were also included to evaluate the environment’s effect on their state of mind—Have you been feeling optimistic about the future? Have you been feeling good about yourself? Have you been feeling relaxed? Lastly, the interviews captured participants’ opinions about UGS and their role in improving well-being and quality of life.

## 5. Results: Experiences and Insights

A total of 242 photographs and two videos were received over the course of the qualitative research, surpassing the minimum requested. The solicited photographs and diaries served as the basis for the interviews. Each interview was personalized to delve into the participant’s documented experiences. Following Braun and Clarke’s [68] approach to evaluating qualitative data, the photographs were analyzed through thematic analysis, categorizing them by recurrent themes. All UGS photographs were included for examination in the semi-structured interviews, as well as photographs that included natural elements, which was another theme. The discussion below covers the key observations concerning each of the preconditions for green space utilization defined by Van Herzele and Wiedemann [14], their effect on well-being, and quality-related observations. The participants’ photographs illuminated how the importance of these preconditions shifted for them during the pandemic. They also revealed the well-being inequalities caused by differences in these preconditions.

### 5.1. Results: Access

Thirteen of the 16 participants reported having access to a UGS within walking distance—20 min was the longest reported walking time (Participant 6). The three participants with no access belong to lower-income quintiles (quintiles 1 and 3), which is indicative of how the city’s spatial inequalities are linked with neighborhoods’ socioeconomic levels [32]. An additional four participants reported losing access to a nearby UGS due to the closure of some green areas because of COVID-19 restrictions. Notably, none of the participants without UGS access near their home indicated that they belonged to the two highest income quintiles.

Participants who lacked access to a green or open space during the pandemic reported that they did not use any UGS or waited long periods to travel to a UGS outside their neighborhood. For example, Participant 16 revealed that she visited an urban forest located on the city’s outskirts every two to three weeks. To reach the UGS, she had to use public transportation. She reported that every time she took the bus, she felt anxious due to the risk of contagion, which partially mitigated the positive effect of the UGS on her well-being:

“I used to take the bus to go see my daughter twice a week, and we went to the park in front of her house. Now, my husband and I try to go to the forest outside the city every two or three weeks. It’s good for our health to walk a little bit, but since it is far, we can’t go very often…I’m afraid to use buses a lot since they’re quite packed.”

**Figure 1:**
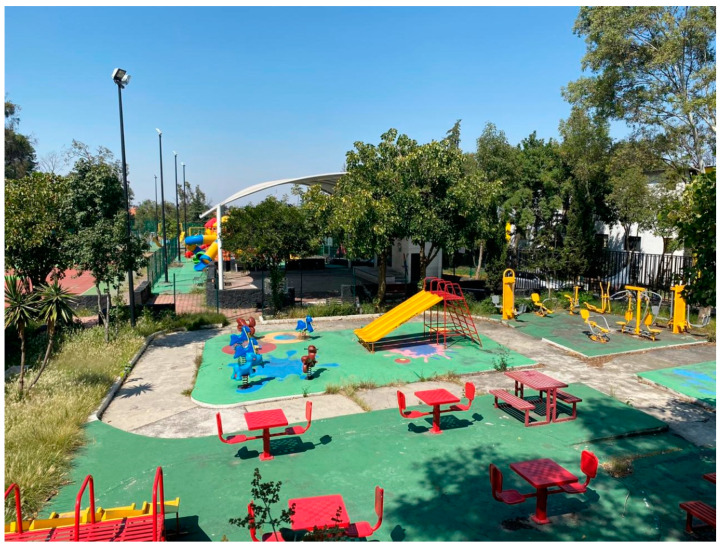
Park near Participant 5’s home. The area was recently renovated by neighbors and closed due to COVID-19 restrictions.

Additionally, six of the seven participants without access to UGS reported other consequences of this deficiency: reduced physical activity and feelings of stress about not having a place to unwind. Such was the case for Participant 5, who stated that he did not exercise at all because his neighborhood UGS had closed (see Figure 1), and he lacked sufficient space at home:

“Before COVID, I went out more often with my colleagues, my friends. I would go to the Historic Center for a walk or shopping. I also regularly exercised on the courts in the park around the corner, but they are closed right now. I can no longer use them…Not being able to de-stress by exercising has affected the way I feel even more.”

In contrast, seven of the nine participants with access to UGS recounted using these spaces for walking, running, or going out with their dogs or children, which improved their moods. Interestingly, the six participants with dogs used green spaces more frequently than the rest of the group, showcasing that having dogs might contribute to spending time outside.

**Figure 2:**
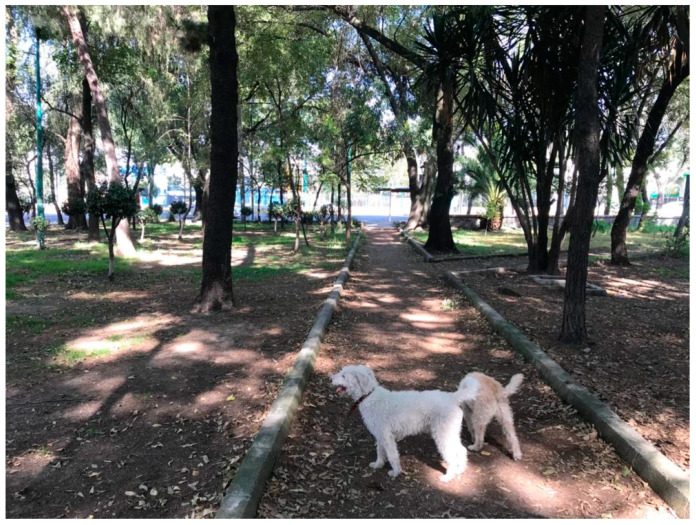
One of the three UGSs near Participant 1’s home.

Participant 1, who had access to a park, described using it to walk his dogs and unwind from tensions at home (see Figure 2). The number of hours that the participant’s family spent together had increased significantly since March, which generated tensions among family members. The participant stated that going for a walk in the park allowed him to relax and think more clearly:

“Right now, I feel like I have more time, and I don’t know how to handle it anymore. I want to do a lot of things, but I am locked up. I don’t want to spend so much time in front of the computer or sitting down. I want to go out and not feel so secluded. Going to the park helps me to think with greater clarity.”

### 5.2. Results: Size

UGS size played an essential role during the pandemic. For example, several small public areas were closed due to density concerns. As mentioned above, four participants experienced the closure of UGS in their neighborhoods. These participants explained that the closures were connected to the small size of the spaces, which could not support high occupancy rates. Furthermore, six participants mentioned that they had heard announcements in the media encouraging citizens to exercise at home or in large open spaces, emphasizing avoiding small, crowded areas.

Before COVID-19, Participant 2 used the park near her home to play basketball. However, when public health officials decided to close it, she was left with no other option but to exercise at home. This had a negative impact on her physical well-being and health:

“I miss playing basketball. I feel like I have gained weight because of the lack of exercise. The park where I played every week is closed. They told us that it was too small to guarantee that people wouldn’t be close to each other. Now, I don’t know how to exercise at home.”

When asked about the importance of size for using UGS in ordinary circumstances, six participants indicated that size was not an issue as long as the space had the facilities to practice their sport of choice, such as basketball courts or soccer fields.

**Figure 3:**
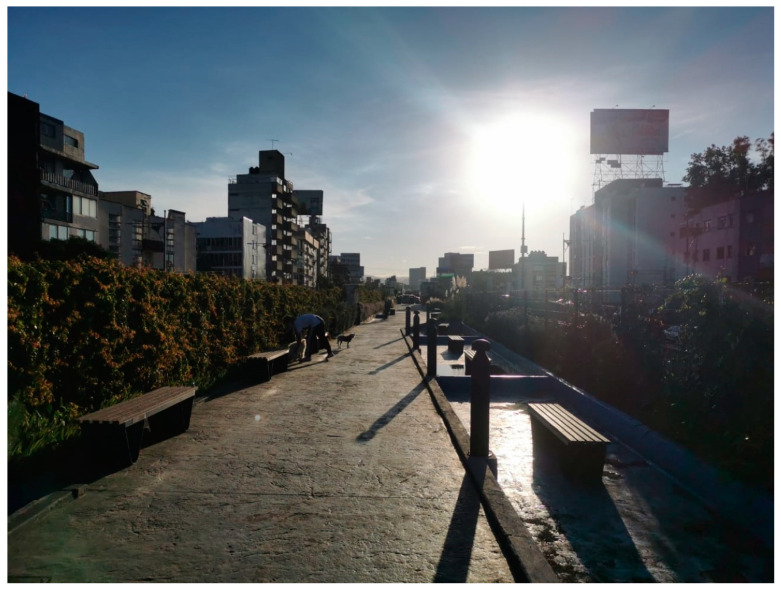
Park near Participant 8’s house. The park’s occupancy rate is monitored by police.

While size influences the functionality of green spaces, it had little effect on the use of areas that remained open during the pandemic. Participants noted that small, public green areas in their neighborhoods remained open and consistently attracted users. This was the case for Participant 8, who belonged to the fourth income quintile and frequently used the small UGS near her home (see Figure 3). Notably, the space has no grass or trees, only vertical walls. The participant mentioned that the space was constantly monitored to ensure that the number of users did not exceed the capacity indicated by the authorities. This gave her peace of mind while going out for daily walks with her baby. Participant 8’s experience also shows that, beyond size, there were inconsistencies in park closures across neighborhoods with different socioeconomic statuses—high-income boroughs experienced fewer restrictions:

“Since the pandemic started, I only go out once a day to walk my baby in the park two blocks from my house. My daily walks with my baby make me feel free, relaxed. Although the park I go to is small, I like it because there are police officers ensuring that there are not many people.”

**Figure 4:**
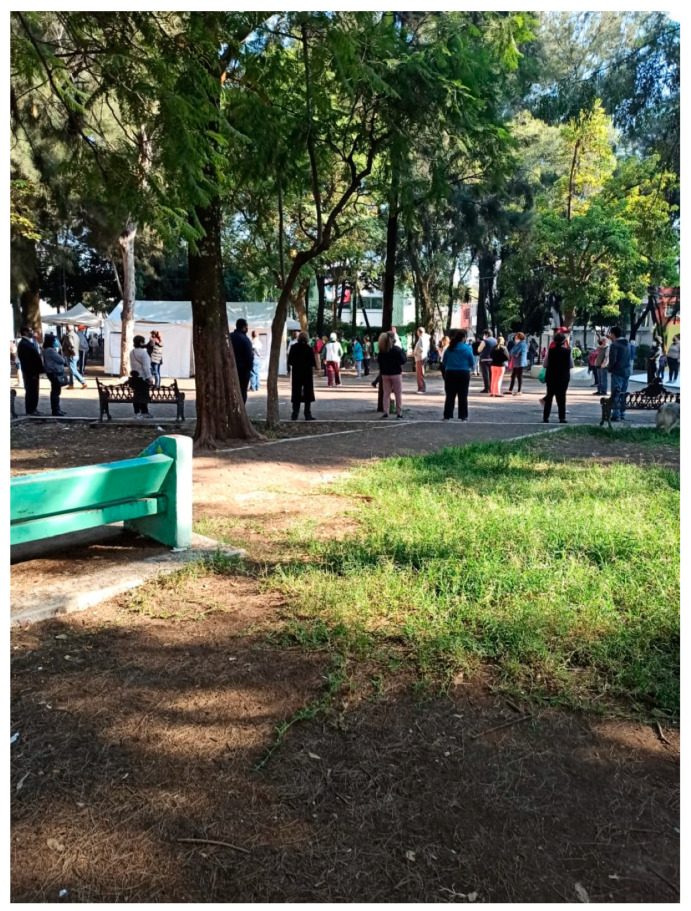
Testing center inside a park near Participant 11’s apartment complex.

Often, open areas with the physical capacity for social distancing were eventually used as COVID-19 testing sites. This became an additional limitation on the use of these spaces. For example, despite having access to what he described as a medium-sized park, Participant 11 revealed that he felt uneasy using this green space. The municipal government had installed a testing center there, forcing him to avoid the UGS except to pass through it (see Figure 4). The other green area near Participant 11 is located inside his apartment complex. Since the space is relatively small, opportunities to exercise there are limited. Nonetheless, he mentioned that looking at the greenery through his window positively affected his mood despite the size of the space.

### 5.3. Results: Safety

The results reiterate the presence of the two dimensions of safety discussed above: violence and health. Every female participant expressed some degree of anxiety related to violence, regardless of the quality of the UGS in their neighborhood. In contrast, no male participants mentioned any safety concerns related to violence when using UGS. Some of the women interviewed revealed feeling even more unsafe during the pandemic due to the lack of people, both in the streets and at UGS—this led to the fear of not being heard while screaming for help during an attack. For instance, Participant 7 indicated that although she had access to a large UGS near her home, she did not use it because she would not feel safe doing so. She preferred to run in the streets around her house in a gated community. Likewise, Participant 13 reported that she did not use the UGS in her neighborhood, as this park is known to host gang activity, and she feared she might be attacked.

It should be stressed that the fear of violence was present among participating women of all socioeconomic levels. Participant 10, who belongs to the highest income quintile and lives in a high-income neighborhood, echoed participants’ sentiments from the lowest quintiles. She described feelings of anxiety when walking through a large UGS due to the possibility of being assaulted. She also voiced that she avoided going to large UGS alone and visited a small park close to home to walk her dog. She only used the large UGS when her male partner could accompany her. Like the park located near Participant 8’s home, the park next to Participant 10’s home remained open despite its small size.

When asked about her perception of safety at UGS, Participant 9’s concerns had exacerbated. She referred to the number of femicides in Mexico (11 per day), indicating that since the beginning of the pandemic, UGS tended to be less populated, making her feel even more at risk while alone:

“Since I live alone, I can’t go out much for fear of something happening to me. If I go out, it’s with my brother who sometimes visits me. After all the disappearances of women this year, why take the risk?”

All the female participants agreed that improved lighting would make them feel safer and increase their willingness to use UGS more often. In addition, some mentioned the presence of the police as a sign of safety. However, this was not the case for all participating women. Three participants—2, 10, and 16—expressed the opposite opinion, stating that they felt uncomfortable in police presence; five more did not mention the police at all. Previous studies have suggested a shared perception in Mexico that the police are associated with corruption or criminal activity [69]. Notably, these three women belong to income quintiles 1, 2, and 5, which suggests this concern is not contingent on economic status. Participants 5 and 11, who are male, also shared this opinion. For example, Participant 11 said:

“Today, in the parking lot of a commercial plaza, I could see some patrols of the national guard (militarized police). The presence of police groups with military weapons makes me feel nervous, tense. I do not feel safer around a police group of this nature.”

**Figure 5:**
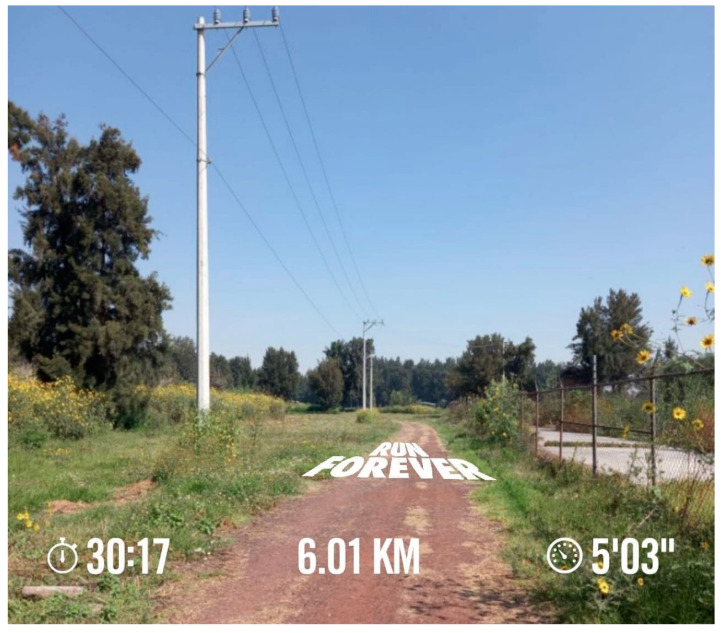
Bosque de Tláhuac, UGS near Participant 2’s apartment complex.

While only female participants expressed fears about violence, both men and women voiced health concerns. Male participants who used UGS (Participants 1, 6, and 14) indicated feeling safer while using large UGS due to their ability to distance themselves from others or avoid people altogether, thus reducing contagion risk. For example, Participant 14 articulated a sense of calm when using the UGS “Desierto de Los Leones”, a large urban forest in the city. He even mentioned feeling free because he did not have to wear a mask while exercising because there were no other people nearby. Participant 6 commented that he started a new routine to improve his health (see Figure 5). The routine included running every other day in the Bosque de Tláhuac, a large UGS located a 20-min walk from his home. This UGS was limited to 30% of its regular capacity, making him feel comfortable using the space without fears of contracting COVID-19. Despite noting that the space lacked adequate lighting and the bathrooms were in poor condition, he declared feeling better both physically and mentally due to his new running habit:

“Since the pandemic started, I’ve been able to exercise well. I can exercise with no fear in terms of the risk of contagion because the place I go running, Bosque de Tláhuac, is huge, and there are hardly any people. I’ve lost a lot of weight. I am finally the weight I want to be.”

Interestingly, Participant 6 belongs to the lowest income quintile, while Participant 14 belongs to the highest. Although the men are on opposite sides of the income spectrum and photographs showed their accessible UGS have quite disparate characteristics, both feel safe while using them.

In addition to the men who voiced opinions about health safety, participating women also expressed concerns about infecting relatives or becoming ill themselves. For instance, Participant 15 said she worried about letting her two teenage sons use the park near her home. She noted that most people in the park did not wear a face mask, making her feel uncomfortable. Nonetheless, she feared that her children were becoming apathetic and irritable, so she permitted them to ride their bikes from 6 to 8 AM, thus avoiding unnecessary crowds. Regular exercise during off-peak hours has improved the children’s tempers and alleviated Participant 15’s anxiety:

“I have two teenagers; it is challenging for them because they have nowhere to release all their energy. They miss going out, seeing their friends. I am afraid of letting them go out, but, at some point, I have to.”

### 5.4. Results: Quality

Analysis of the photographs showed that participants’ UGS use during the pandemic occurred independently of quality-related characteristics, even though these features were highlighted as very important in the interviews. Some participants explained this in part by noting that other exercise and recreation venues closed their doors to the public, limiting their options. Under normal circumstances, a variety of alternatives are available, and the quality characteristics cited by the participants take on additional importance as satisfiers.

**Figure 6:**
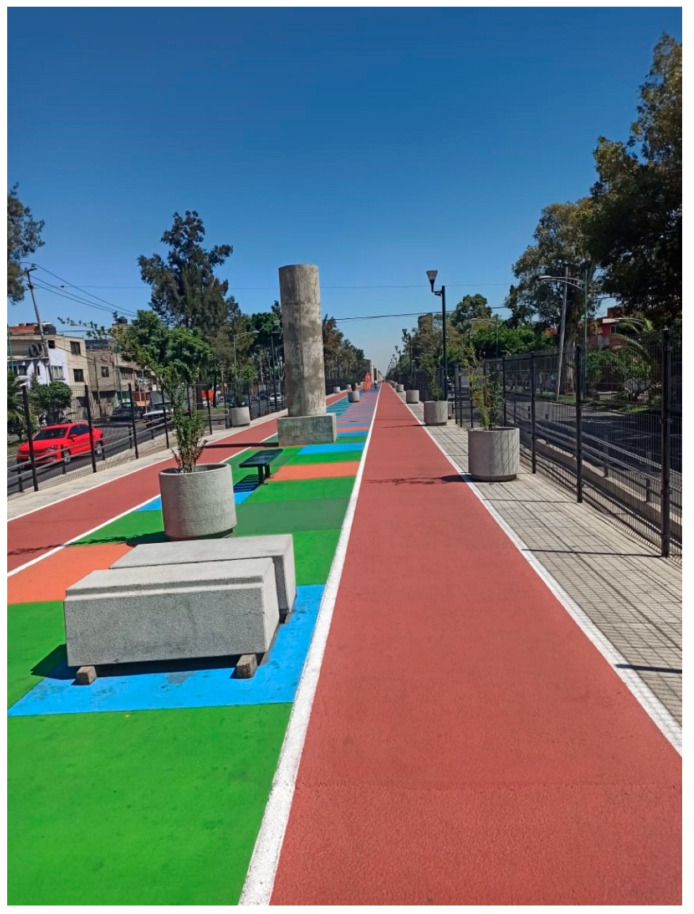
A space identified as a ‘park’ by Participant 11 (Iztacalco).

**Figure 7:**
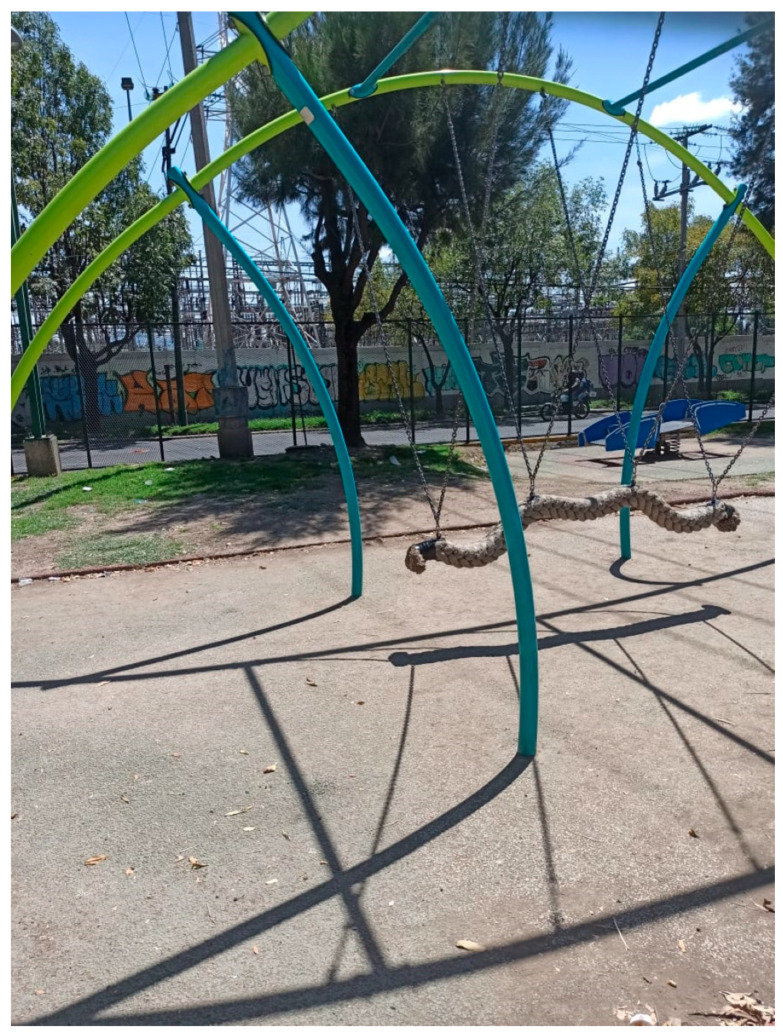
‘Park’ near Participant 5’s home (Álvaro Obregón).

All participants agreed that greenery, such as trees and vegetation, was the main quality satisfier for encouraging UGS use. However, not all the images provided by the participants showed spaces that would traditionally be considered ‘green’. Four lower-income participants (income quintiles 1 and 3) sent pictures of non-traditional UGS. For example, Participant 11 sent a picture of what he defined as a park (see Figure 6). The photograph shows a running track with small potted trees. Participant 5 also sent a photograph of a park located in his neighborhood with neither grass nor trees. The trees seen in the image (Figure 7) are located outside the bounds of the park. Only one upper-income participant, Participant 8, provided a photograph of a non-traditional green space (see Figure 3).

**Figure 8:**
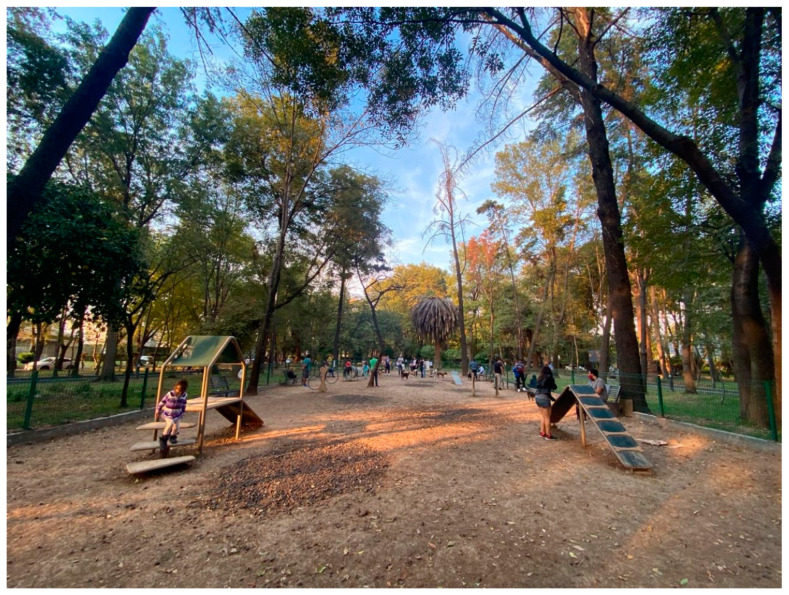
One of the three UGS near Participant 1’s home.

Fourteen out of the 16 participants indicated that green spaces should also provide residents with opportunities to engage in physical activity. Of these 14 people, 11 cited playgrounds or infrastructure specifically designed for exercise as a quality satisfier encouraging use. Six mentioned that specific sports facilities, such as basketball courts, soccer fields, or running tracks, also made UGS attractive for use. However, only six participants provided photographs of parks with built-in features for physical activity or play (see Figure 8).

As previously mentioned, all the women who participated agreed that ample lighting—associated with feelings of safety and comfort—was an essential quality satisfier for UGS use. Only Participant 13 mentioned the presence of local markets as a quality satisfier. This was surprising considering the historical importance of open markets in Latin America [70] and the numerous markets typically located in parks Mexico City parks. Similarly, only Participant 1 raised the importance of community activities for promoting UGS use.

### 5.5. UGS Use and Well-Being

During the interviews, participants were asked to rate the importance of UGS in increasing urban quality of life on a scale from 1 to 10, with 1 being the lowest score and ten being the highest. All participants awarded scores of at least 8. Notably, participants with children or pets said that proximity to parks was a key factor influencing their residence choice. Additionally, three participants—5, 6, and 16—expressed their desire to move to a ‘greener’ neighborhood but said they were unable to do so because of economic constraints.

Furthermore, UGS use appears to serve as a coping mechanism to decrease the effects of stress and isolation caused by the pandemic and increase overall well-being. For example, Participant 1 commented that his neighborhood walks allowed him to identify neighbors who used the space at the same time he did; they greeted each other from a distance, which helped enforce a sense of community.

The seven participants who used UGS regularly reported experiencing positive feelings such as comfort, happiness, and tranquility during their use. UGS users also noted that green spaces’ positive effect on their well-being was intensified due to the pandemic: during months when the traffic light system was set to red, UGS were one of the few options available for spending time away from home, exercising, or relaxing. Overall, participants who used UGS reported feeling physically healthier compared to the group that did not use these spaces. Individuals in the group who did not use UGS described more frequent sensations of anxiety and stress. The latter group comprised residents of low-income neighborhoods and women with safety concerns.

**Figure 9:**
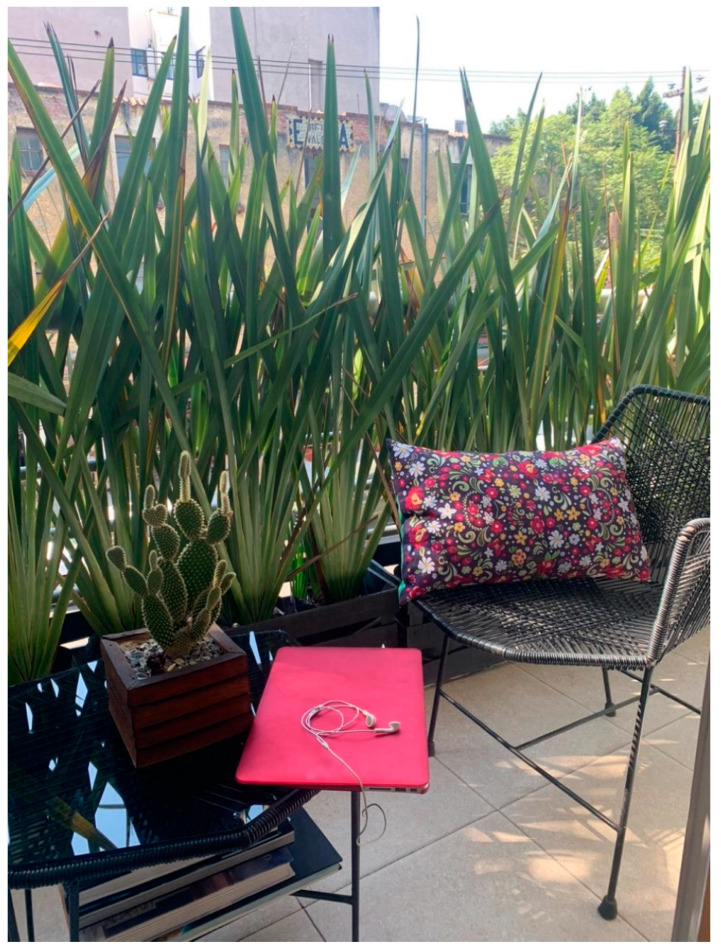
Outdoor patio inside Participant 4’s house.

Beyond UGS, the photographs also depicted other aspects that positively influenced participants’ sense of well-being. Remarkably, 15 out of the 16 participants expressed that contact with nature, such as trees, plants, or flowers, brought them positive emotions that increased their mental well-being. Additionally, 27% of the photographs received (66) included natural elements or were taken in a green space. Participant 4, who did not have local access to UGS, created her own green space on an outdoor patio at home to cope with stress (see Figure 9). She indicated that the area added a sense of calm and helped her to work more productively. She started meditating in March, revealing that the greenery inspired her to do so.

Participant 13, who expressed that the fear of being attacked or robbed inhibited her use of the UGS in her neighborhood, mentioned that looking at the sky and trees from her window made her feel more relaxed and that the fresh air made her feel more serene. She usually used other UGS located in the city center, such as Chapultepec Park; however, she had no private transport means. Using public transport caused her stress due to the risk of contagion.

Meanwhile, Participant 7 could not use UGS due to the extensive travel time needed to reach the closest green space. Nonetheless, she indicated that she felt happy walking on her street due to some improvements that the neighbors had made—local artists had painted some planters along the road. Participant 7 said that incorporating plants into the street scenery was an effective way to upgrade a neighborhood. Notably, both participants who indicated improvements in their neighborhoods, 5 and 7 (income quintiles 1 and 3), highlighted that these were improvements collectively made by neighbors.

**Figure 10:**
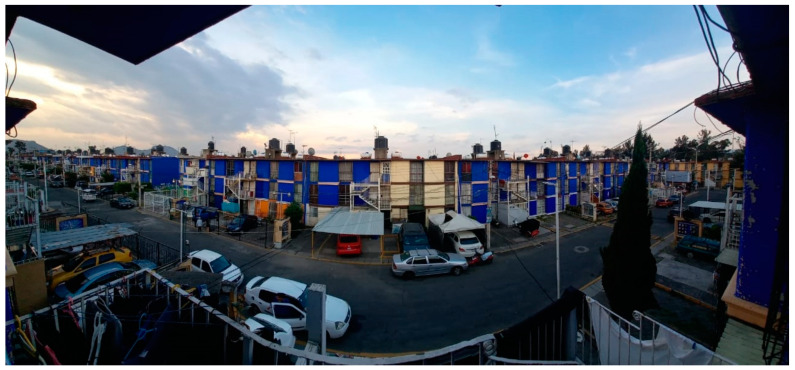
Apartment complex where Participant 6 lives.

Experiencing nature as a coping mechanism was a recurrent theme amongst participants. Even individuals who had access to a large UGS, like Participant 6, expressed a desire for additional contact with nature, such as having trees nearby (Figure 10):

“Today, I did not leave the house at all, and my house is small and offers few and unpleasant views. The landscape full of houses is sad. The bars seem to be a metaphor for the confinement. Would I prefer to see some trees or mountains? Of course, but here we are.”

All participants except two indicated that either a family member, a friend, or an acquaintance had contracted COVID-19; in seven cases, they had died. Those who had experienced loss reported finding comfort by spending time in natural environments or seeing elements of nature. In fact, contact with nature also alleviated other anxieties beyond health-related stress. For example, Participant 2 highlighted that observing the landscape of trees and mountains outside her home helped her feel more relaxed, despite the poor circumstances in which she found herself:

“The notebook on the left side is from the last semester, and the one on the right [is] from my current semester. There is a huge difference between them because, in the last semester, I had everything I needed for my education. Financially, neither my family nor I had problems, which is currently not the case. The pandemic caused my dad to lose his job. I have a hard time concentrating on my studies because I constantly think about the expenses my family carries. I had to sell several material things to get out of trouble, including my laptop, a tool that is important for my education. Going out and looking at the landscape, the trees, gives me a small sense of relief, of hope.”

## 6. Discussion

In this qualitative study, it was observed that the preconditions of green space utilization described by Van Herzele and Wiedemann [14]—access, size, and safety—were all factors affecting the participants’ use of UGS in Mexico City during the COVID-19 pandemic. Throughout the interviews with participants, access or the lack thereof was the most cited precondition for enabling or discouraging UGS use. This can be partially explained by the mobility restrictions imposed by health authorities during the pandemic; most participants stated that they felt inclined to carry out their daily activities within or near their homes. The participants in this study expressed that spending more time outside increased their risk of contracting the virus. Moreover, especially for participants in lower-income groups who often rely on public transportation, the use of buses or the metro led to greater anxiety. Therefore, public transport was largely avoided as a means to reach UGS.

This study’s observations are in line with the results from Fernández-Álvarez [32], which suggest that differences in UGS access are linked to municipalities’ socioeconomic status. Overall, Mexico City’s uneven green space distribution has promoted social segregation and exacerbated the city’s health inequalities [31]; this has become even more evident during the pandemic. The closure of UGS to contain the spread of the virus has disproportionately affected residents of low- and middle-income neighborhoods, who already had limited access to these spaces [32]. Notably, participants living in high-income neighborhoods did not report UGS closures. Considering that UGS use is strongly associated with health, ensuring accessibility for vulnerable populations is essential to maintaining their well-being [71]. The latter has been shown by a previous survey conducted in nine urban parks of Mexico City, and the authors’ conclusions stress the significance of access to UGS for facilitating a sense of well-being [18]. In the same study, Ayala-Azcárraga et al. concluded that residents with better access to UGS use these spaces more frequently, which positively correlates with life satisfaction and supports this project’s results.

While access is a definitive precondition in determining use [17], the evidence in this study indicates that size only became a precondition when it turned into a constraint for keeping spaces open, thereby preventing their use. As mentioned earlier, some municipalities in Mexico City closed small UGS due to the difficulty of enforcing low occupancy rates. However, for the study participants, size per se was not a determinant of UGS use. The latter was revealed in the interviews, in which participants expressed their willingness to use UGS regardless of size. Residents’ disposition to use UGS of all sizes has important urban planning implications, highlighting that the need for urban UGS is not subject to specific acreage conditions.

The current evidence from around the world regarding UGS size as a precondition of use is inconclusive. In a study in Los Angeles, Loukaitou-Sideris et al. [72] found that young users were attracted to parks of different sizes, with recreational activities being the most significant determinant of use. Meanwhile, in a study conducted in Perth’s metropolitan area, Giles Corti et al. [48] revealed that residents were more willing to use larger UGS, as people felt more inclined to walk in more expansive spaces. The present study suggests that access to UGS in certain areas of Mexico City is so limited that size loses relevance as a determinant of use.

Safety in terms of violence had a gender component, with all participating women expressing anxiety. Although some women stated that they would be more inclined to use UGS if police were present, other female participants, as well as two men, expressed distrust and fears toward the police, especially if militarized. On the other hand, all the female participants in this study voiced feeling more likely to use well-lit UGS. This remark is of utmost importance, as it reveals that the quality characteristic of ample lighting may not be a satisfier but, rather, a precondition among women.

Regarding health-related safety issues, both male and female participants expressed concerns about using UGS due to fears of contracting COVID-19 as a result of the lack of people wearing face masks or the difficulty of social distancing. However, not all participants who expressed fears stopped using these spaces. Some participants reported using UGS as a coping mechanism to deal with the stress of confinement, which outweighed the health risks. Relatedly, the results of an online survey of mental well-being distributed in nine countries during the pandemic revealed that contact with green spaces and nature improves mental well-being, demonstrating that this effect is stronger than the anxieties associated with health safety [73].

While all but one of the participants suggested that the quality of UGS was an extremely important satisfier for use, those who used UGS during the pandemic restrictions did so regardless of whether the spaces met their quality standards. Moreover, all participants who were users reported positive feelings while using UGS regardless of quality. Nonetheless, it remains unclear whether this would hold up under normal circumstances when people have more recreational options available and are keen to travel long distances. Previous research in Latin American cities, including in Mexico, has suggested that urban residents prefer to use areas with quality characteristics such as ample vegetation, numerous benches, playgrounds, cleanliness, and regular maintenance [18,74]. Nonetheless, this study’s participants defined areas with little vegetation as parks and were willing to use them regularly. Additional research is needed to explore the relationship between the quality and use of UGS under normal circumstances.

Overall, participants in this photo-journal study revealed an increased awareness of the importance of using UGS to maintain their physical and mental well-being, particularly under COVID-19 restrictions. For instance, parents relayed concerns about the limited physical activity in their children’s daily routines. They stated that their children’s health improved to some extent thanks to UGS, which Guan et al. [75] have described as a global concern. Some participants even reported feeling that the benefits of using UGS intensified given the pandemic circumstances, which has also been reported in countries like Spain [73]. Importantly, this study illustrates that participants benefited from exposure to nature (whether it be to plants, flowers, or trees), associating it with increased mental well-being.

## 7. Limitations and Strengths

The present qualitative study has three main limitations. First, the results are specific to Mexico City and, therefore, cannot be generalized to other settings. However, it is reasonable to argue that some observations might be similar to experiences in other places. For example, evidence from an online survey distributed worldwide showcases the increased awareness of UGS’s importance for physical and mental well-being since the pandemic started [73], which could lead to a stronger association between UGS use and well-being. The latter highlights the need to improve UGS access. Moreover, safety concerns in terms of violence, which have a strong gender component, are also shared in other Latin-American cities and signify additional barriers for use for women [76]. Thus, it could be expected that better lighting in UGS would increase UGS use for women in other Latin-American cities. Further qualitative evidence is needed to test these hypophyses and assess the extent to which the results of this study are relevant to megacities in other countries.

Second, it is worth noting that this study did not include older adults in the sample of participants. This is a selection bias limitation, resulting from contacting participants through social media, with older adults being less inclined to use it [77]. In Mexico City, existing inequalities in UGS access might have also decreased the UGS use of the elderly in low-access neighborhoods, as has been the case in other countries. For instance, a recent study conducted in the UK with older adults revealed that people above 65 were less likely to use UGS than middle-aged people, with movement restrictions worsening UGS access for the 65+ group [78]. Another study in Vancouver, Canada, showed that UGS use influenced the perceived physical and mental well-being of the elderly [79]. However, health safety concerns and limited access to UGS during the pandemic reduced these spaces’ therapeutic impact. Further qualitative research is needed to adequately capture the perspectives of the elderly in Mexico City, avoiding recruiting participants with the use of digital tools and social media.

Finally, the duration of this study is relatively short. Since emotions and perceptions vary in short periods, it would be extremely valuable to compare these results with participants’ perceptions later on or conduct a more prolonged study [60]. We will address this limitation by conducting a second phase of the study to inquire about participants’ behaviors and emotions after a year of the implementation of COVID restrictions in Mexico City.

As far as strengths, this study’s main advantage is its use of triangulation of data to understand UGS use and its effect on well-being from different perspectives. With the combination of photography, diaries, and semi-structured interviews, it is possible to evaluate both verbal and visual data [80], providing an additional layer of analysis on how the environment influences human well-being. This study not only describes use patterns as its predecessor but is critical in addressing the reasons for using or avoiding using UGS during the pandemic. Notably, conducting these types of studies can aid in developing and validating quantitative instruments [56].

Another strength is that the study evaluated daily recollections of participants’ actions and emotions, which has proven to be more accurate than asking for remembered experiences when capturing and understanding feelings and life assessments [60]. Lastly, as previously mentioned, participants in this study had no supervision and could take pictures of what they considered important. This minimized the authors’ influence on participants’ behavior and eliminated the possibility of an authoritative researcher–researched relationship [81].

## 8. Conclusions

The present qualitative study results cannot be generalized but offer important snapshots of the behaviors and experiences of a specific group of residents in Mexico City. The research contributes to the emerging literature on the incentives or deterrents to UGS use during the COVID-19 pandemic and its possible implications on well-being. The observations suggest that UGS could be used as a public health tool to create opportunities for improving well-being in urban areas. These spaces serve as equalizers for people who otherwise lack access to health resources.

Interviews with participants seem to indicate that the heterogeneity in UGS distribution (more than their size) should be considered in the city’s urban development plans. The lack of access to UGS has shown most detrimental for participants in low-income neighborhoods. Additionally, this study’s findings suggest that including elements that heighten safety perceptions could make these spaces more inclusive. The perception of inadequate safety in UGS related to the threat of violence is an obstacle that seems to affect women primarily. Comprehensive policies aimed at intersectoral collaboration encompassing park improvements and public safety are needed to build healthy communities and increase UGS use among Mexican women.

Urban planners and policymakers must consider the importance of UGS and natural elements as public health opportunities, bearing in mind the potential long-term savings in health and social care costs. The results suggest that increasing the presence of natural elements such as trees, plants, or flowers in urban design is essential to promote better mental well-being among the population of Mexico City. Furthermore, citizen participation instruments, such as the present study, can be used to incorporate residents’ perspectives into urban design and policymaking. Such inclusion is crucial to optimizing the use of public spaces and building a healthier city.

## Figures and Tables

**Table 1:** Participant profiles.

Participant	Age	Gender	Municipality	Quality of UGS in the Neighborhood	Living Situation	Income Quintile
1	22	Male	Coyoacán	4	Living with family	4
2	22	Female	Gustavo A. Madero	3	Living with family	2
3	24	Female	Cuauhtémoc	2	Living with roommates	3
4	25	Female	V. Carranza	3	Living with family	3
5	26	Male	Álvaro Obregón	1	Living with family	2
6	26	Male	Tláhuac	2	Living with family	1
7	26	Female	Tlalpan	2	Living alone	3
8	32	Female	Benito Juarez	4	Living with family	4
9	32	Female	Benito Juarez	4	Living alone	3
10	33	Female	Miguel Hidalgo	5	Living with a partner	5
11	35	Male	Iztacalco	3	Living with family	2
12	37	Female	Benito Juarez	4	Living with roommates	3
13	44	Female	V. Carranza	3	Living with family	2
14	45	Male	Cuajimalpa	5	Living with family	5
15	56	Female	Azcapotzalco	3	Living with family	3
16	58	Female	Milpa Alta	1	Living with a partner	1

## Data Availability

The data is not meant for public use.
